# Non-Coding RNAs in Retinal Development

**DOI:** 10.3390/ijms13010558

**Published:** 2012-01-05

**Authors:** Nicola A. Maiorano, Robert Hindges

**Affiliations:** MRC Centre for Developmental Neurobiology, King’s College London, New Hunt’s House, Guy’s Campus, London, SE1 1UL, UK; E-Mail: nicola.maiorano@kcl.ac.uk

**Keywords:** retina, microRNA, non-codingRNA, visual system, neurodegenerative diseases, posttranscriptional inhibition, regulatory network, development

## Abstract

Retinal development is dependent on an accurately functioning network of transcriptional and translational regulators. Among the diverse classes of molecules involved, non-coding RNAs (ncRNAs) play a significant role. Members of this family are present in the cell as transcripts, but are not translated into proteins. MicroRNAs (miRNAs) are small ncRNAs that act as post-transcriptional regulators. During the last decade, they have been implicated in a variety of biological processes, including the development of the nervous system. On the other hand, long-ncRNAs (lncRNAs) represent a different class of ncRNAs that act mainly through processes involving chromatin remodeling and epigenetic mechanisms. The visual system is a prominent model to investigate the molecular mechanisms underlying neurogenesis or circuit formation and function, including the differentiation of retinal progenitor cells to generate the seven principal cell classes in the retina, pathfinding decisions of retinal ganglion cell axons in order to establish the correct connectivity from the eye to the brain proper, and activity-dependent mechanisms for the functionality of visual circuits. Recent findings have associated ncRNAs in several of these processes and uncovered a new level of complexity for the existing regulatory mechanisms. This review summarizes and highlights the impact of ncRNAs during the development of the vertebrate visual system, with a specific focus on the role of miRNAs and a synopsis regarding recent findings on lncRNAs in the retina.

## 1. Introduction

**Figure 1:**
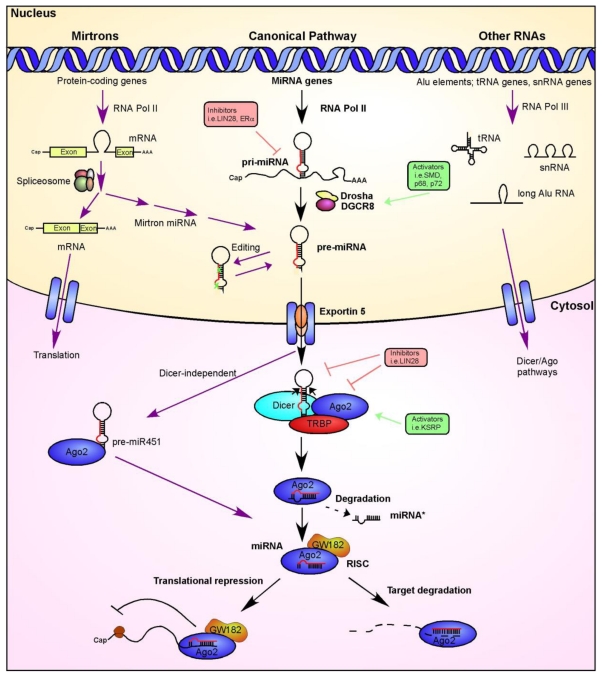
MicroRNA biogenesis pathway.

During the development of the nervous system, a sophisticated interplay between different molecules in an organism takes place to generate the correct cell types at the correct time, allow them to migrate to the appropriate places and finally connect to each other in a proper way to ensure normal functionality. One of the major challenges has been the characterization of the regulatory network underlying these fundamental steps. The visual system, apart from the interest as a sensory system *per se*, has been one of the prominent models to help characterizing general molecular mechanisms in neural development. The eye develops from an initial lateral evagination of the diencephalon and a subsequent formation of the optic cup [1]. Retinal cells differentiate in a clear chronological order from a population of multipotent retinal progenitors to generate the seven principal cell types [2,3]. The correct establishment of axial polarity of the retina ensures that retinal ganglion cells (RGCs), the only projection neurons of the eye, form synaptic connections with different brain targets in an appropriate retinotopic manner [4]. And finally, individual classes of retinal cells can be further divided into sub-types that connect to each other within function-specific circuits [5]. To date, many protein-encoding genes have been identified, which ensure the correct development of the eye and the establishment of connectivity within the retina and with other targets in the nervous system. In addition to protein-coding mRNAs however, and different from the well-characterized RNA molecules which perform infrastructural and housekeeping roles (such as rRNAs, tRNAs, and snRNAs), a large number of so-called non-coding RNAs (ncRNAs) were found to be expressed in the developing central nervous system [6–8]. Emerging data suggest that the enormous number of ncRNAs, which have little or no protein-coding potential, contains most of the information that control the expanded regulatory framework in eukaryotes, increasing the complexity of such organisms [9]. Intensive studies in the last decade led to a distinct classification of ncRNAs, mainly according to aspect of origins, structure and biological functions. Selected from these different categories, we will discuss here microRNAs (miRNAs) and long ncRNAs (lncRNAs). MiRNAs represent a large family of endogenous 21–23 nucleotide-long RNAs, which regulate gene expression mainly through post-transcriptional regulation [10]. The generation of miRNAs in a cell has long been viewed as linear and universal to all mammalian miRNAs, following therefore a “canonical” pathway (Figure 1). However, recent studies have identified additional features that do not obey this simple miRNA maturation pathway. Furthermore, it has been shown that the metabolism and function of miRNAs is in turn subject to a sophisticated control ([11] and Figure 1). This multistep control and the complexity of the different pathways have to be carefully taken into account in order to appropriately interpret results from experimental perturbations of the system.

MiRNAs interact with their mRNA targets through direct Watson-Crick base-pairing of the 5′ region of the mature miRNA—known as the seed sequence—and the 3′UTR of partially complementary mRNAs. This enables the presence of combinatorial effects with other miRNAs and RNA-binding proteins that associate with the same target mRNAs. Considering that miRNAs can regulate large numbers of targets [12,13] they represent a fundamental feature during the global control of spatial-temporal changes in a given cell or organ [14,15]. In addition to identifying the function of individual miRNAs, an approach that has been widely used is the removal of the ribonuclease Dicer, one of the critical enzymes for miRNA maturation and thus representing a bottleneck in the biogenesis pathway [16]. Early studies using this approach showed that miRNAs have a critical role during brain development, including eye formation [16]. The subsequent generation of a conditional allele for Dicer in mice allowed the removal of this protein, and thus the majority of miRNAs, in a specific spatial-temporal fashion using the Cre-Lox technology [17]. Although this method faces some limitations, the different conditional Dicer-deletions that have been generated in the retina resulted in important contributions to the field.

In eukaryotic cells most of the miRNAs are generated through the “classical” canonical pathway (Figure 1, middle section, black arrows). However, studies carried out in the last few years highlighted several exceptions due to the presence of modulators of this canonical pathway, including inhibitors or activators (Figure 1, red and green squares) or alternative pathways (left and right sections, purple arrows). In the canonical pathway (Figure 1, middle): MiRNA genes are transcribed by RNA polymerase II to give rise to long primary transcripts (pri-miRNAs), which form hairpin structures. This step is subject to regulation by transcriptional activators or inhibitors (not shown). In the nucleus pri-miRNAs are then processed by a protein complex containing the RNase III Drosha, resulting in a 60–70-nucleotide stem/loop structure with a 3′ overhang (pre-miRNAs). This step can be modulated by activators (green box) and inhibitors (red box) that act on the RNA stem/loop structure or on the Drosha activity. Furthermore, in some cases, pre-miRNA sequences can be subject to base modifications by an editing process. The pre-miRNAs are then exported to the cytosol with the help of Exportin-5, through a GTP-dependent process. There, the pre-miRNAs are further processed by the Dicer-TRBP complex to yield a 21–24 nucleotide long miRNA/miRNA* duplex molecule. Also this step is subject to regulation through inhibitors and activators, acting on the complex and the stem/loop structure. Finally, mature miRNAs are loaded into an RNA-induced silencing complex (RISC) that includes Argonaute (Ago2). An exception has been reported for pre-miR-451, which can follow a Dicer-independent pathway and is directly loaded into the RISC-Ago2 complex after processing by Drosha. The miRNA then binds to the 3′ UTRs of specific mRNA targets and depending on its seed sequence, leads either to translational repression or to the cleavage and degradation of the target. In the mirtron pathway (Figure 1, left): Pre-miRNAs can be also generated through an alternative, Drosha-independent pathway. Here, miRNA sequences are located in introns of primary transcripts generated by RNA polymerase II. Their expression pattern is therefore exactly following the mirtron-harboring gene. The miRNA sequence is subsequently spliced out from the primary transcript by the spliceosome and enters then the sequence of the canonical pathway. Other RNAs (Figure 1, right): Exceptions to the canonical pathway have been also found for transcripts generated by RNA polymerase III. They are first processed in the nucleus in a Drosha-independent manner and then transported to the cytosol where they may be subject to a Dicer/Ago-dependent pathway.

Long ncRNAs represent a class of RNA molecules that is fundamentally different from miRNAs in their function and in the way the act. In contrast to the short sequences of miRNAs, the transcripts for long ncRNAs are usually longer than 200 nt and can even reach more than 100 kilobases, as shown for macro ncRNAs [18]. They are one of the most abundant classes of ncRNAs transcribed in the genome, being highly expressed in neural tissues [19,20]. Similar to protein-coding genes, the transcription of lncRNAs can be dynamically regulated showing specific temporal and cell-type specific expression [21,22]. Furthermore, the primary transcript can also be post-transcriptionally modified, including 5′ capping, 3′ polyadenylation and splicing, as known for conventional mRNAs [9,23]. As a consequence of their increased length and sequence, the transcripts of lncRNAs are able to form specific secondary structures with clear functional features [24,25]. They have been shown to control the cellular gene expression program at multiple levels [26]. However, in contrast to miRNAs, only a small number of functional lncRNAs has been described to date. One of the major functions appears to be the regulation, both in *cis* and in *trans*, of the epigenetic status of proximal and distal protein—coding genes through the recruitment of chromatin-remodeling complexes [27,28]. However an individual lncRNA can act, in turn, at several levels of gene expression making it difficult to attempt a simple classification [26]. In the developing retina the expression of several lncRNAs has been described, even though the characterization of clear functions remain largely to be elucidated [6,29,30].

Here, we provide an overview of recent advances in understanding the role of ncRNAs during visual system development and function. In the first part we focus on the family of miRNAs, summarizing the studies that have been carried out to profile their expression pattern in the vertebrate retina and the experiments aimed at their functional characterization during normal retinal development. Finally, we also discuss the emerging roles of lncRNAs in the retina.

## 2. Identification of miRNAs in the Retina

Many large-scale efforts applying different strategies, such as small RNA isolation followed by cloning or deep sequencing and small RNA library sequencing, have been undertaken to discover miRNAs in a variety of species, including in mammalian genomes [31,32]. The number of individual miRNAs identified has therefore been steadily increasing and ranges in the thousands today [33–36]. In particular, there are currently more than 1500 human miRNA and over 800 mouse miRNA sequences deposited in the miRbase database [37,38]. One of the first studies that included the mouse eye as a source tissue identified 8 specific miRNAs [39], whereas a systematic study in zebrafish identified at least 15 miRNAs in the eye [40]. These early studies showed that a considerable number of miRNAs are expressed in a tissue-specific or even cell-type specific manner. It is therefore not surprising that several laboratories have consequently analyzed the miRNA transcriptome in the retina. Some of these studies used qPCR or cloning approaches and therefore reported simple miRNA expression profiles, without analyzing the expression patterns in more detail [41–43]. However, with the development of more applicable hybridization techniques for small RNA probes, some reports started to include also retinal expression patterns [44–47] in addition to studies using different retinal laminae isolated by laser capture microdissection as a source [48]. Interestingly, comparing data from different expression profiling reports, it became clear that a particular miRNA cluster (miR-183/96/182) was specifically expressed not only in the retina [43], but also in the inner ear [49] and the dorsal root ganglion [50], suggesting a “sensory-organ specific miRNA cluster” [43]. Further investigation showed that this cluster is indeed also expressed in the olfactory and tongue epithelia and that the genes for these three miRNAs are located within a 4 kb genomic region on mouse chromosome 6qA3 and are transcribed as a single polycistronic pri-miRNA, an arrangement conserved from *C. elegans* to human [43,51]. It was found that the members of this cluster miR-183/96/182 are all expressed in rods, cones and bipolar cells, whereas other miRNAs follow different, lamina-specific expression patterns, for example miR-181a in RGCs and amacrine cells [45,46,52] or let-7d in bipolar and amacrine cells [52].

Based on the early miRNA expression profiling reports from mouse, human [39] and zebrafish [40], the Banfi laboratory described initially a set of 7 eye-specific miRNAs, including their spatial expression pattern in the retina [45]. A particular advance compared to other studies was the inclusion of different developmental stages revealing changes in cell and lamina-specific expression of certain retinal miRNAs over time. The same group increased their efforts using a large-scale approach that resulted in the generation of a miRNA expression atlas of the mouse eye, named miRNeye [53]. This fully searchable atlas contains high-resolution images of in situ hybridizations from over 200 miRNAs at 4 different stages (E16.5, P0, P8 and P60). It is therefore a valuable resource in order to study possible functions of miRNAs in the eye from development to adult.

## 3. Function of MicroRNAs in the Visual System

MicroRNAs have been identified to play important roles in almost all biological processes, from early neural developmental events, including neurogenesis or cellular differentiation up to later aspects such as synapse maturation or function [54,55]. One of the main challenges however still in the field is to attribute clear roles to individual miRNAs. An obvious complication is the high number of candidate target genes that can be found for each miRNA using different bioinformatics software, such as TargetScan, miRanda or PicTar [56–58]. In addition, these algorithms produce only predictions of possible targets and the actual binding of a miRNA to a 3′UTR of a candidate target mRNA has still to be confirmed empirically. A second difficulty is that miRNAs generally rather “fine-tune” the protein output of a cell and therefore a loss of function phenotype for a single miRNA may be very subtle [12,13]. Finally, the highly specific regulation of the miRNA maturation pathway and the variation of the degradation rate for each miRNA increase the variability and the complexity of targeted gain and loss of function studies [11]. Many laboratories therefore tried to overcome these difficulties through a complete (or at least substantial) deletion of miRNAs, in order to study the general role for these molecules in a particular system or pathway.

### 3.1. Dicer Deletions

Several studies to date have used this approach to delete Dicer in a spatial-temporal fashion the visual system. As discussed above, Dicer is a ribonuclease III and one of the essential enzymes for the maturation of most miRNAs (Figure 1). Therefore, the deletion of Dicer leads to a lack of such miRNAs and enables the analysis of a system in the absence of most miRNAs. However, it is important to note that there are Dicer-independent pathways to generate functional miRNAs ([11] and Figure 1) and that studies using Dicer deletions do not show a 100% loss of all miRNAs. The straight deletion of Dicer in mice leads to an embryonic lethal phenotype at E7.5 mostly due to a depletion of stem cells in the embryo [59]. The generation of a conditional Dicer allele in mice made it possible to circumvent these problems and delete Dicer by crossing with lines that express the Cre-recombinase under the control of specific promoters [17].

The first report analyzing a conditional Dicer deletion in the retina used the Chx10-cre line [60]. This BAC-transgenic mouse line harbors a Cre-GFP cassette under the control of the Chx10 promoter [61] and leads to a mosaic expression of the Cre-recombinase in the retina. As a consequence, retinal cells (although not all of them) lack functional Dicer protein [60]. The authors reported that retinae from these mutant mice showed normal lamination and no alteration in the generation of the different retinal cell types during embryonic and early postnatal development. However, at later stages, the mutant mice generated photoreceptor rosettes that had been described previously in pathological retinal conditions, associated with degeneration and/or abnormal proliferation of retinal cells [62]. Further analysis showed no increase in proliferation and that the phenotype was attributed to a degenerative phenotype. After postnatal day (P) 16, the mutant retinae became increasingly affected by degeneration, showing a higher number of rosettes and disorganization of the retinal laminae. In parallel, the mutant mice showed abnormalities in their ERG responses probably as a result of the retinal degeneration [60]. The relatively late onset of apoptosis after Dicer deletion was surprising, since studies carried out in several other systems before and since then have shown an almost immediate cell death phenotype upon loss of Dicer [17,63–65].

A different study in the same year reported the downregulation of Dicer in the *Xenopus* retina by using morpholinos that were electroporated into retinal progenitor cells [66]. Here, the Dicer knock-down led to photoreceptor rosettes and increased retinal cell death similar to the phenotypes seen in the Chx10-cre; Dicer^fl/fl^ mice. In addition however, the eyes appeared to be overall smaller in some morphants with strong defect in retinal lamination. Furthermore translation of some of the genes involved in controlling retinal cell fate, such as Xotx5 and Xotx2, was delayed, prompting the authors to suggest that miRNAs are controlling the cell clock machinery for timing the retinal neurogenesis [66].

A more detailed analysis on the generation of different retinal cells upon Dicer deletion came from the Reh laboratory [67]. Here, the floxed Dicer allele was used in combination with a Pax6-α-cre line, that expresses the Cre-recombinase in the nasal and temporal distal portions of the retina starting at around E10.5 [68]. In contrast to the earlier study in mouse [60], the authors detected aberrancies already starting at E16. Using a number of retinal cell-type specific markers it was shown that the deletion of Dicer leads to a overproduction of early generated retinal cell populations, such as RGCs and horizontal cells, and on the other hand, a downregulation of late progenitor cells [67]. Probably as a consequence of the latter, the Dicer-deleted areas of the retina show a decrease of the late generated cell types (amacrine cells and rod photoreceptors). These results suggest that Dicer (and therefore probably miRNAs) has an important function in the regulation of progenitor competence. In addition, a general increase in apoptosis was detected from early developmental stages onwards, which extends postnatally so that by P8 all the Dicer—negative cells have disappeared [67]. Finally, the authors found that the retinal progenitor cells in Dicer mutant mice failed to express the transcription factor Asc1, a major regulator of Notch signalling components, suggesting that this pathway may be—at least in part—responsible for the detected phenotypes. This hypothesis was subsequently confirmed by a recent study from the same authors showing that Dicer mutant retinae exhibit a decrease in Notch signalling [69]. Indeed, by crossing the conditional Dicer mice into a mouse line that constitutively expresses the Notch intracellular domain—therefore generally increasing Notch signaling—they observed a restoration of normal horizontal cell numbers. However, other major phenotypes, such as the increased generation of RGCs or the competence of retinal progenitor cells were not rescued, suggesting that the loss of Notch signalling in Dicer mutants has only consequences for specific retinal cell types [69]. Changes in the Notch signalling components (and Hedgehog components) upon Dicer deletion were previously reported in a parallel study using the same Pax6-α-cre driver in combination with the floxed Dicer allele [70] to generate a retina-specific loss of function. Here, the authors investigated the role of Dicer in the formation of anterior structures of the optic cup and specifically the generation of neural and non-neural tissues during eye development. In addition to the α-cre line, the authors used two other Cre-lines, the Tyrp2-cre and Pou4f3-cre to delete Dicer in pigmented retinal cells and in postnatal non-pigmented ciliary body plus the iris pigmented epithelium, respectively. This resulted in a general increase of apoptotic cells in Dicer-deficient regions, consistent with the findings from earlier studies. Further investigation of the mutant mice showed an aberrant patterning of the distal portions of the optic cup, where usually neural and non-neural progenitor cells reside to create the two different compartments, the neural retina and the ciliary body. The mispatterning in the Dicer mutants resulted in a loss of a clear border between these compartments and furthermore, defects in iris and ciliary body formation were detected upon loss of Dicer [70].

A different aspect of visual system development was analyzed in a study by Pinter and Hindges [64], which focused on RGC axon outgrowth. The visual system has for a long time been one of the major systems to investigate neural circuit formation, including axon pathfinding decisions at the midline, topographic map formation, axon branching and synaptogenesis [4,71,72]. Here, the authors used a mouse line that expresses the Cre-recombinase under the control of the Rx promoter [73]. The retinal homeobox (Rx) gene is an eye field transcription factor that is essential for eye formation [74] and its expression starts at around E7.5 in the anterior neural plate. As a consequence, conditional alleles are deleted in all cells that lead to the development of the retina from the start of eye field specification. The study showed that mutant mice exhibited severe micropthalmia, detectable from the time of eyecup closure onwards, combined with a wave of apoptotic cells peaking at E13.5. Interestingly however, the overall structure of the eye, including the formation of the cup and the closure of the optic fissure was normal, suggesting that miRNAs and Dicer are not involved in these early morphogenic processes [64]. RGCs were found to send out axons correctly through the optic disc to form the optic nerve. However, in the optic fiber layer inside the retina, as well as in the optic tract at the ventral hypothalamus, it was apparent that the RGC axons are defasciculated. The detection of a general lack of adhesion upon Dicer deletion is consistent with other findings showing that Dicer is essential for the adhesion of epithelial cells in the eye or lung and in kidney cell types [70,75,76], suggesting a general role for miRNAs for this function. The main phenotype concerning axon pathfinding however, was seen at the optic chiasm, with a aberrant segregation between ipsi- and contralateral projections, combined with a high number of axons turning at the midline and growing into the contralateral eye, as well as some axons extending abnormally into the diencephalon [64]. These phenotypes were not the result of a mispatterning of the eye (or the chiasm), suggesting that miRNAs have direct functions in intracellular processes needed for axon growth and pathfinding. However, Dicer-negative RGC axons are able to initially innervate correctly their major targets, the superior colliculus and the lateral geniculate nucleus (Maiorano and Hindges, unpubl. obs.) and further experiments are ongoing to investigate the role of Dicer in retinotopic map formation.

A similar early conditional deletion of Dicer was described recently, where the authors use a mouse line in which the coding region of Foxg1, a transcription factor expressed from E8.5 onwards in the mouse telencephalon and optic vesicle, has been replaced by the Cre-recombinase [77]. Again, a high rate of cell death was reported in the developing retina, in addition to micropthalamia associated with depigmentation and the absence of the lens. However, here the phenotype is more complicated to dissect and to contribute uniquely to the action of miRNAs (or Dicer), since the Cre-positive cells in the lens and the nasal portion of the eye also loose at least one functional copy of Foxg1 [78].

The most recent report using the conditional Dicer allele in combination with a mouse line that expresses Cre in retinal progenitors from around E10.5 onwards (Dkk3-cre) showed again a microphthalmia phenotype, coupled with an increase in apoptosis and abnormal differentiation of several cell types, in line with previous reports. *In vitro* re-aggregation experiments demonstrated that these effects are cell-autonomous [79].

In summary, these data resulting from the conditional deletion of Dicer have lead to multiple interesting phenotypes discovering possible functions of miRNAs in retinal development and cell maintenance. One has to, however, take some caution. We are not yet at a point to fully understand all the functions of Dicer in the cell and therefore cannot automatically attribute all phenotypes seen in Dicer mutants exclusively to miRNA function. More work has to be done in the analysis of individual miRNAs, miRNA families, or pathways specific to miRNAs in order to get unambiguous data. This point is supported for example by findings from a recent study investigating the molecular basis for geographic atrophy (GA), an advanced form of age-related macular degeneration (AMD) where cells of the retinal pigmented epithelium (RPE) degenerate [83]. The authors detected lower levels of DICER1 in RPE cells of humans with GA. Dicer deletion in mouse RPE cells by expressing the Cre-recombinase under the RPE-specific BEST1 promoter (using an Adeno-associated viral vector) in a Dicer^fl/fl^ background lead to specific cell degeneration. A first possible conclusion was that the lack of miRNAs is responsible for the degenerative effects. Further analysis however showed that this was not the case, since the injection of the same AAV-BEST1-cre vector into other mouse models where different genes essential for the miRNA biogenesis pathway were conditionally targeted, including Drosha^fl/fl^, Dgcr8^fl/fl^ or Ago2^fl/fl^ mice [84–86], did not result in similar RPE degeneration. Surprisingly, Dicer was found to have a completely different function, namely in protecting the RPE cells from toxic RNA elements. It was shown that primary transcripts from the Alu elements present in the human genome (B1/B2 RNA in mice) are inducing cell death in the RPE and that in the normal situation Dicer cleaves these approx 300 nucleotide-long sequences into small, non-toxic fragments. Dicer down-regulation in GA therefore leads to an accumulation of Alu RNA (or B1/B2 RNA in mice) that induces cell death [83]. As discussed above, all studies to date using conditional Dicer deletions reported a large increase in apoptosis in the affected tissue. Considering the data from the AMD model, it remains to be seen if cell death in other tissues than the RPE, is indeed exclusively a consequence of the lack of miRNAs or if Dicer here also—at least in part—plays a wider role, for example through clearing the cells of toxic RNA. Interestingly, possible additional roles of Dicer were already pointed out in the first report of a retina-specific Dicer deletion [60]. These results make a strong case for using other approaches in addition to Dicer-deletions to globally delete miRNAs in order to analyze unambiguously their function in a system, for example by using different conditional deletions affecting the miRNA pathway.

### 3.2. Functions of Individual miRNAs in the Visual System

Only a few reports exist where the role of a one particular or a small group of miRNAs have been investigated in the visual system. In *Xenopus*, a set of miRNAs, miR-129, miR-155, miR-214 and miR-222, was identified that targets the otx2 and vsx1 genes and leads to their translational inhibition. These two genes are essential to specify late-born bipolar cells in the retina [87–89] and although transcribed already in early progenitors, their translation is not detected until later stages. The authors found that the cell-cycle dependent expression of the four miRNAs is needed to inhibit early translation of these homeobox transcription factors until the appropriate time to generate bipolar cells [90]. A different study performed in *Xenopus* investigated the role of the brain-specific miR-124 during retinal development [91]. Although a reliable down-regulation of miR-124 could not be achieved, the overexpression of this miRNA resulted in an elongated retina coupled with a shorter optic stalk and an inhibition of retinal cell proliferation. As a possible direct target, Lhx2 was identified [91]. The down-regulation of an other miRNA, miR-24, in the *Xenopus* eye lead to increased apoptosis and a small eye phenotype, whereas its over-expression was sufficient to prevent cell death [92]. RNA-binding assays identified the pro-apoptotic factors caspase9 and apoptosis protease-activating factor 1 (apaf1) as direct target genes, suggesting that miR-24 acts as an important regulator of cell death during retinal development by repressing an apoptotic program [92].

In mouse, a study analyzed the genetic deletion of miR-182, a miRNA highly abundant in the retina that belongs to the miR-183/96/182 cluster discussed above [93]. Given its strong expression in the retina, one would have predicted a clear phenotype upon deletion of the gene. However, the resulting miR-182 mutant mice did not reveal any structural abnormalities in the retina during development and throughout the first weeks after birth [93]. Furthermore, no significant changes in global gene expression could be detected in the miR-182 loss of function mice, using microarray profiling. It remains to be shown if these mice show any physiological phenotypes in the retina.

Finally, we summarize here some recent exciting findings that investigated the role of specific miRNAs not directly related to a function in the retina, but rather in the general visual system. The first study came from the Filipowicz lab [94] and performing a detailed investigation on the miR-183/96/182 cluster and on miR-204 and miR-211, two miRNAs located in introns of two genetic loci encoding cation channels. The miR-183/96/182, mostly expressed in photoreceptors, and the miR-204/211 enriched in the cells of the inner nuclear layer showed a highly dynamic profile of expression, being up-regulated in light condition and down-regulated upon dark adaptation. Interestingly, the authors showed a surprisingly fast turnover of miRNA molecules in the order of an hour, against the previous general view of a very slow miRNA turnover in cells [95]. The rapid turnover was shown to be activity-dependent and resulted from a fast decay coupled to a fine regulation of Pri-miRNA transcription upon light adaptation. In turn they regulate neuronal activity through a pathway involving the voltage-dependent glutamate transporter SLC1A1 and the sodium/potassium transporting ATPase ATP1B3 [94]. Interestingly, the results in this study suggest not to be visual system specific, but rather represent a general mechanism for activity-dependent turnover rates for neuronal miRNAs, enabling fast changes in mRNA translation for example in dendritic spines.

Two recent reports described the crucial role of miR-132 in the plasticity of the mouse visual cortex during the “sensitive period” shortly after eye opening [96,97]. Independently, they showed that pri-miR-132 transcription is regulated at epigenetic levels by activity-dependent mechanism, activated upon light induction. Through *in vivo* gain-and loss-of-function approaches using miR-132 mimics [96] and miR-132 sponges [97] the authors showed a recovery of plasticity and synaptic development concerning binocular area of visual cortex and a prevention of ocular dominance shift, respectively, in mice with monocular deprivation. These experiments showed that light- (*i.e.*, activity) induced miR-132 levels are crucial for ocular dominance plasticity during the critical period in visual cortex.

## 4. MicroRNAs and Retinal Diseases

Studies over the last decade have clearly established a link between miRNAs and diseases within and outside the nervous system [98,99]. A particular focus has been the involvement of this family of non-coding RNAs in neurodegeneration [100], where the majority of data available is based on either linkage analysis of mutations in miRNAs or their 3′UTR target binding sites and the disease or simply on profiling miRNAs in different diseases. In the retina, studies have been shown that different mouse models of retinitis pigmentosa (RP) exhibit altered miRNA expression profiles [52,101]. The authors found that all the members of the miR-183/96/182 cluster are down-regulated in RP mice, whereas miR-1, miR-133 and miR-142 are up-regulated. Computational analysis has resulted in a long list of possible direct and indirect targets for these miRNAs, but the clear mechanisms how the small RNAs lead to the retinal degeneration remain to be elucidated. Similar profiling experiments have been done in hypoxia-induced retinal and choroidal neovascularization, because of the link of these processes to several diseases including age-related macular degeneration and diabetic retinopathy [102]. The authors identify seven miRNAs that are substantially increased and three that are substantially decreased in these mouse models. Injection of pre-miR-31, -150 or -184 significantly reduced retinal neovascularization, and injection of pre-miR-31 or -150 significantly reduced choroidal neovascularization, although no clear link to possible target genes has been established *in vivo* so far [102].

As discussed already above, in a specific form of age-related macular degeneration a causal effect has been demonstrated for Dicer, but surprisingly in relation to a miRNA-independent function [83]. Therefore changes in miRNA profiles in a particular disease can obviously be due to a secondary effect and not represent the underlying cause for the development of the disease. More work is needed to establish the causal relation between miRNAs and the disease. However, independent of this, the possibility remains to use such expression profiles as biomarkers.

## 5. Long Non-Coding RNAs in the Visual System

Several studies in the last decade have lead up to the identification of more than a thousand long non-coding RNAs (lncRNAs) [103] some of which are specifically expressed in the nervous system [104]. They are functionally clearly distinct from the miRNAs discussed above. Many lncRNAs are also subject to a specific post-transcriptional modification such as splicing, polyadenylation and 5′ capping, similar to conventional mRNA processing [105]. A difference to the shorter miRNAs is that lncRNAs can control every level of the gene expression pathway, such as chromatin modification and transcriptional control in addition to post-transcriptional regulation [19]. Such modulation can be carried out through *cis*- and *trans*-acting mechanisms [106]. Recently a database has been developed grouping together information from several lncRNAs, including some expressed in the retina [29].

In most cases that have been characterized for the developing retina, lncRNAs share the 5′ *cis*-regulatory element with protein-encoding genes but they are transcribed in a head-to-head divergent way. Here the lncRNAs are named natural antisense transcript (NAS) or opposite sense transcript (OST) [30,107]. Many retinal homeodomain factors that are critical during retinal development have NAS/OST associated with their transcriptional unit, including Six3, Pax6, Six6, Vax2, Otx2, Pax2 and Rx [107,108]. Studies on such NAS/OST revealed the presence of many isoforms suggesting a detailed transcriptional and post-transcriptional regulation. Some of the isoforms are also translated, however the functions of the resulting proteins are unknown [107]. The NAS/OST expression pattern can either perfectly overlap with [109] or can be absolutely different from [107] the partnered protein-coding transcript. In several cases perturbation of protein transcript levels affected also the partnered NAS/OST level of expression as seen for Vax2 [107]. A study by the Blackshaw laboratory described that for 100 transcription factors expressed during retinal development, no less than 35 are NAS/OST associated [30]. Considering the vast data on lncRNA expression, it is surprising that only in a few cases the function of lncRNAs have been characterized. Recently, the Banfi laboratory performed an elegant functional study on the activity of the opposite strand transcript Vax2os during retinal development [110]. From the 5 different isoforms present, the authors focused their study on the strongest expressed isoform Vax2os1. They first showed that Vax2os1 expression, similar to the sense Vax2 transcript, is localized in the ventral retina during mouse embryogenesis and strongly down regulated during postnatal stages. However, unlike Vax2, Vax2os1 is mainly expressed in the outer neuroblastic layer (oNBL) and is again upregulated in adult animals in the ventral outer nuclear layer (ONL). Through functional analysis using a gain-of–function approach, the authors demonstrated that overexpression of Vax2os1 in retinal proliferating neuroblasts results in an alteration of normal cell cycle progression of the photoreceptor progenitor cells toward their final differentiation. In particular, they demonstrated a delay in the differentiation of the photoreceptor progenitor cells upon Vax2os1 overexpression. This therefore suggests that lncRNAs can act as regulators of cell cycle progression during cell differentiation. However, further studies need to be performed in order to identify in detail the molecules that interact with such lncRNAs and the underlying mechanisms.

Another example, although in a model different from the retina, is Evf-2, a lncRNA associated with the Dlx5/6 locus (encoding two homeodomain transcription factors expressed in retinal progenitors). Through an interaction with DLX2, another Dlx family member, Evf-2 can regulate in *trans* the Dlx5/6 transcription [111]. A recent study characterized the Six3 opposite strand transcript (Six3OS) in the developing mouse retina [112]. The data shows that Six3OS plays an important role in retinal cell specification. Furthermore, using gain- and loss-of-function experiments, the authors illustrate that Six3OS does not control Six3 expression levels but rather regulates Six3 activity, suggesting an interaction in *trans*. Binding studies suggest that Six3OS acts through the recruitment of histone modification enzymes to Six3 target genes [112]. Several possible interactions in trans between lncRNAs and their own or different loci have been hypothesized for the retina, but are still subject to investigation [30]. Additional evidence of genetic regulation by lncRNAs in *trans* outside the retina exists for example in Drosophila, where ncRNAs of trithorax response elements recruit epigenetic modulators to the specific location of ultrabithorax [30,113,114].

Other cases where lncRNAs may regulate related protein-coding genes in *cis* through a mechanism of transcriptional facilitation or interference, have been described in yeast, where for example the Serine-mediated expression of the intergenic transcript SRG1 represses an adjacent gene SER3, which is involved in serine biosynthesis [115], and in pituitary cells where a 5′ locus control region activates the transcription of adjacent genes [116]. These results leave the possibility for similar *cis*-mechanisms present also in the retina [30].

In addition, lncRNAs that are not located near protein-coding genes are also expressed in the retina. For example the lncRNA Tug1 has been identified as a regulator of rod photoreceptor development, even if the exact mechanism has not yet been characterized [117]. Furthermore, the twin nuclear localized lncRNAs Xist and Tsix (regulators of X chromosome inactivation) are expressed in a subset of retinal cells in the outer and inner neuroblastic layers during development, keeping a low expression level in some RGCs and photoreceptor cells upon differentiation. However, neither the role nor a possible correlation with the X-chromosome inactivation pathway of Xist/Tsix in these cells has been clarified [108]. The retinal non-coding RNA 2 (RNCR2), expressed widely in developing nervous system, represents 0.2% of all polyadenylated RNAs in the neonatal retina. RNCR2 negatively regulates amacrine differentiation and development of Muller glia cells. Nevertheless, also in this case, the underlying molecular mechanism remains unclear [118].

In the future, the use of high-throughput sequencing and microarray hybridization approaches in combination with functional *in vitro* and *in vivo* studies should shine some more light on the role of lncRNAs during retinal development and functional maintenance.

## 6. Conclusion

Considering the relatively short time since the initial discovery of non-coding RNAs, the studies to characterize their function have resulted in a large amount of novel data and successfully pushed the field forward. However, new findings also show that the system is probably more complex than initially thought. In the visual system we have made steady progress in characterizing miRNAs and lncRNAs and linking them to function, although for many there is still a gap present regarding their precise action or their actual target genes. For the future, it will be both challenging and exciting to discover much more about the ncRNAs involved in the complex regulatory mechanisms to generate a fully functional retina, including its connectivity and physiological properties.

## Figures and Tables

**Table 1:** Conditional Dicer deletion studies in the mouse visual system.

Cre-line	Onset of Creexpression	Location of Cre-expression (visual system)	Survival (homozygotes)	Reported phenotypes	Reference
Chx10-cre [61]	E10.5	Retinal progenitors, mosaic	Normal	Photoreceptor rosettes, apoptosis, abnormal ERG responses	Damiani *et al*., 2008, [60]

Rx-cre [73]	E7.5	Eye-forming tissues, anterior neural plate	Die at P0	Micropthalmia, apoptosis, axon guidance defects	Pinter & Hindges 2010, [64]

Pax6-α-cre [68]	E10.5	Nasal and temporal distal regions of the retina	Normal	Micropthalmia, apoptosis, increase of early retinal cells, decrease of late progenitors	Georgi & Reh, 2010, [67]
				Detached iris pigmented epithelium, hypoplastic ciliary body	Davis *et al*., 2011, [70]

Tyrp2-cre [80]	E9	From E11 in pigmented retinal cells, at late embryogenesis in presumptive pigmented epithelia of the ciliary body and the iris and in the muscles/stroma of iris; (detectable from E9 onwards in eye, forebrain and DRGs);	Poor at adult stages	Microphthalmia, most iris tissues missing, hypoplastic ciliary body	Davis *et al*., 2011, [70]

Pou4f3-cre [81]	P1	Non-pigmented ciliary body and iris pigmented epithelium	Die soon after birth (most)	Detached iris pigmented epithelia, hyperplastic ciliary body	Davis *et al*., 2011, [70]

Foxg1-cre [78]	E8.5	Optic vesicle, lens, telencephalon, olfactory epithelium, ear, foregut, isthmus	Die just before birth	Microphtalamia mostly associated with high apoptosis and depigmentation in nasal retina, lens missing.	Kersigo *et al*., 2011, [77]

Dkk3-cre [82]	E10.5	Retinal progenitors	Die 4–6 weeks postnatal	Micropthalmia, apoptosis, defects in retinal cell differentiation	Iida *et al*., 2011, [79]
